# Clinical Applications of the Cone Contrast Test in Ophthalmology and Neurology

**DOI:** 10.3390/jcm14093079

**Published:** 2025-04-29

**Authors:** Priya Raju, Minzhong Yu

**Affiliations:** 1 School of Medicine, Case Western Reserve University, Cleveland, OH 44106, USA; 2 Department of Ophthalmology and Visual Sciences, University Hospitals, Case Western Reserve University School of Medicine, Cleveland, OH 44106, USA; 3 Cole Eye Institute, Cleveland Clinic Foundation, Cleveland, OH 44106, USA; 4 Department of Ophthalmology, Cleveland Clinic Lerner College of Medicine of Case Western Reserve University School of Medicine, Cleveland, OH 44195, USA

**Keywords:** cone contrast sensitivity test, quantitative color vision test, inherited color vision deficiency, diabetic retinopathy, macular degeneration, cone dystrophy, cone–rod dystrophy, optic neuritis, glaucoma, traumatic brain injury, non-traumatic brain injury

## Abstract

Color vision is a critical aspect of human visual perception, yet traditional assessments often lack quantitative precision. The Rabin Cone Contrast Test and its successors offer objective, standardized measurements of cone-specific contrast sensitivity. These tests improve the detection and classification of color vision deficiencies and can facilitate the monitoring of color vision deficits in inherited retinal diseases, cone dystrophies, optic neuropathies, and brain injuries. Integrating quantitative color vision testing into clinical practice presents a more reliable, reproducible, and functionally relevant evaluation, highlighting its value in disease diagnosis, characterization, and management.

## 1. Introduction

The human eye can distinguish approximately 2.3 million colors with wavelength differences as small as 0.25 nm, enabled by the 6.4 million cone cells in the fovea [1,2]. While cones are outnumbered by 125 million rod cells, they are crucial for both color vision and visual acuity (VA). Though VA tests measure minute visual angle differences [3], most color vision tests offer only qualitative assessments.

Traditional color vision tests do not precisely identify the affected cone type (L, M, or S) or the severity of color vision deficiency (CVD). Furthermore, only a few tests detect S-cone sensitivity loss. Consequently, individuals with CVD are often misclassified.

Introduced in 1996 and first made commercially available to the U.S. Air Force in 2012, the Rabin Cone Contrast Test (CCT) provides a rapid, computer-based, and quantitative measure of cone-specific contrast sensitivity [4]. It evaluates L-, M-, and S-cone function by displaying colored letters designed to be perceived by a single cone type, using the International Commission on Illumination (CIE) standard observer model while equalizing luminance between the letters and background to eliminate brightness cues. The original letter or Landolt C optotypes are presented at progressively decreasing contrast levels in 0.16 log steps, with cone contrast thresholds ranging from 27.5% to 1% for L and M cones and from 173% to 7% for S cones. Thresholds are converted to a one-hundred-point scale, where each correctly identified optotype counts as five points, providing a standardized and easily interpretable measure of color vision sensitivity [5,6,7].

**Figure 1:**
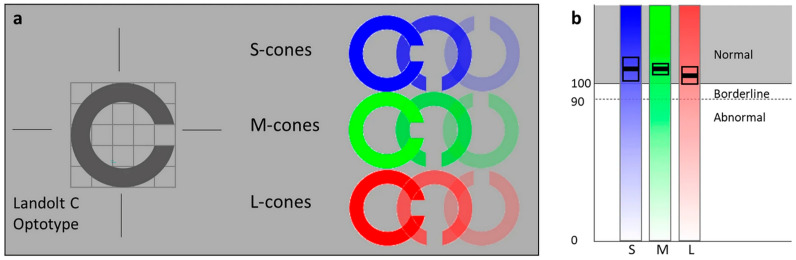
Schematic representation of the ColorDx^®^ CCT HD^®^. (**a**) Participants are shown a series of Landolt-C optotypes, displayed in a randomized orientation with cone contrast against an isochromatic photopic (~74 cd/m^2^) background, and are asked to identify the orientation within 5 s. (**b**) Results show the mean sensitivity score (thick black horizontal line) and standard error (black rectangle) for each cone type. The test uses a Psi-Marginal Adaptive Technique, a Bayesian thresholding method that dynamically adjusts the contrast of the Landolt-C based on the subject’s previous responses to refine in their color vision threshold. The final score is calculated using the Psi-marginal algorithm, which determines the minimum contrast level required for the patient to correctly identify the Landolt-C orientation. Scores range from 175 (indicating best possible color vision) to 0 (indicating the worst color vision). A score below 75 indicates a color deficiency according on the U.S. Air Force’s pass/fail criteria [9].

Successors to the Rabin CCT include the ColorDx^®^ CCTHD^®^ (Color Contrast Threshold for High Definition, Figure 1) and OcuTest Extended Rabin CCT. These tests feature proprietary log contrast sensitivity values, scoring systems, and classification criteria. All CCTs show 100% sensitivity in detecting and classifying red–green (RG) and yellow–blue (YB) CVDs, providing valuable tools for clinical and occupational color vision assessments [8].

This review first outlines traditional and computerized color vision tests, then explores the clinical applications of the CCT in disorders that affect color vision. By providing precise, objective measurements of cone-driven function, the CCT improves our ability to detect, monitor, and manage color vision deficiencies across various clinical contexts.

## 2. Comparison of Color Vision Tests

Color vision testing began in the 1700s and has since evolved into sophisticated computerized techniques for identifying congenital and acquired CVDs [10]. Traditional tests—such as pseudoisochromatic plates, arrangement tests, and the anomaloscope—have played a crucial role in detecting and classifying CVDs, which is essential for early intervention in children, detecting ocular pathology, and evaluating occupational fitness. Computerized tests, including the CCT, Color Assessment and Diagnosis (CAD) Test, and Cambridge Color Test, quantify color perception impairment across all color axes or cone types.

This section reviews both traditional and computerized color vision tests used in clinical settings, focusing on their performance in detecting, classifying, and quantifying CVDs in individuals with presumed normal color vision. All reported performance metrics are based on available study data. A summary of the tests discussed is shown in Table 1.

### 2.1. Pseudoisochromatic Plate (PIP) Tests

These tests use patterns of colored dots discernible only to individuals with normal color vision. The 38-plate Ishihara Test is widely used for RG deficiency screening, with a sensitivity of 98.4% on three errors (*n* = 486), as validated by the anomaloscope [11]. The 14-plate test has a specificity of 100% (*n* = 60) [12]. While effective for quick screenings, the Ishihara Test does not assess YB deficiencies or quantify severity.

The Hardy–Rand–Rittler (HRR) Test detects both RG and YB deficiencies and differentiates between protan (red-deficient) and deutan (green-deficient) anomalies. Its performance metrics vary in each study. Sensitivity ranges from 87% (*n* = 486) to 98% (*n* = 150) on three errors [11,13], while specificity varies from 33% (*n* = 60) to 100% (*n* = 150) [12]. The HRR Test is much longer than the Ishihara Test and is not deemed satisfactory for screening purposes.

In recent years, computerized and smartphone-based adaptations of the Ishihara Test have emerged, offering fast and user-friendly options for color vision screening [18]. While not diagnostic or capable of detecting YB defects, such digital versions provide a practical solution for widespread screening, especially in resource-limited environments.

### 2.2. Arrangement Tests

Arrangement tests, such as Farnsworth–Munsell 100 Hue (FM100), require individuals to arrange colored discs in a gradient. This test is used for distinguishing moderate-to-severe RG deficiencies and has a sensitivity of 81.3% (*n* = 146) to 100% (*n* = 60) and a specificity of 83% (*n* = 60) to 95.4% (*n* = 146) [12,14]. The FM100 has difficulty detecting mild CVD.

The Farnsworth D15 (D15) test is a simplified version of the FM-100, designed for quicker detection of moderate-to-severe CVD. It has a sensitivity of 58% and a specificity of 100% (*n* = 52) [15].

All PIP and arrangement tests are highly sensitive to lighting conditions. Thus, standardized lighting—such as the CIE Illuminant D65, which simulates average daylight at a color temperature of ~6500 K—is often recommended for consistent and reliable testing [19].

### 2.3. Anomaloscope

The anomaloscope is considered the gold standard for diagnosing RG CVD, particularly in distinguishing protan from deutan anomalies. The test requires individuals to match the RG ratio in a light to a reference yellow field by adjusting the intensity and hue. The Rayleigh Equation is used to differentiate between protan and deutan defects, while the Moreland Equation detects blue–green deficiencies, though with lower reliability. It is primarily used for RG deficiency diagnosis, showing 100% sensitivity and specificity in several studies [15,20]. However, its use is limited by the need for extensive examiner training and high costs, making it more common in research settings than in routine clinical practice.

### 2.4. Computerized Tests

Computerized tests offer more precise and quantitative methods for detecting and classifying CVD. The CAD Test presents moving colored stimuli against an achromatic background to independently assess red, green, yellow, and blue thresholds. It shows 100% agreement with the anomaloscope for the detection of congenital RG color deficiencies [21]. A web-based, free downloadable version of the CAD has shown 93.33% sensitivity and 100% specificity [12]; there is a lack of publicly available sensitivity and specificity data for the fully calibrated CAD Test.

The Cambridge Color Test is based on the PIP and similar to the ColorDx^®^ CCT HD^®^. In this test, patients identify the orientation of a Landolt-C optotype presented in a hue that targets a specific color axis. It includes Trivector and Ellipses tests to assess RG and YB discrimination, which are reliable for diagnosing congenital and acquired CVD. There is a lack of statistically significant sensitivity and specificity data for the Cambridge Color Test.

The CCT measures the individual sensitivity of cone types, demonstrating 100% sensitivity and 100% specificity for diagnosing, identifying, and categorizing types of CVD [7,17]. These findings have been validated against the anomaloscope and replicated across several studies [7,8,17].

## 3. Clinical Applications of Cone Contrast Threshold Test

### 3.1. Congenital Color Vision Deficiencies

Trichromatic vision relies on three classes of retinal cone photoreceptors with distinct photopigments: short-wave sensitive (S, blue), middle-wave sensitive (M, green), and long-wave sensitive (L, red). Each photopigment has a specific peak spectral sensitivity (λmax), meaning it is most responsive to light at a particular wavelength. However, the cones’ spectral sensitivities overlap, allowing them to respond to a range of wavelengths. This facilitates the perception of a full color spectrum.

CVD arises from the loss (dichromacy/monochromacy) or alteration (anomalous trichromacy) of the λmax of retinal pigments. M and L photopigment disruptions result in RG CVD, while S photopigment anomalies cause YB CVD. Luminance perception, derived from the ratio of L/M cones, may also be affected in CVD where there is reduced L-cone and M-cone density [22]. Table 2, adapted from the study of Simunovic et al. [23], summarizes the inheritance patterns and prevalence of the congenital CVDs discussed in this section. We will first examine four CVDs caused by mutations in genes encoding cone photopigments, then analyze one color vision disorder characterized by a complete lack of color vision.

#### 3.1.1. Protanopia/Protanomaly (L-Cone Defect)

L-cone opsins, encoded by the OPN1LW gene, typically exhibit a λmax around 566 nm, while M-cone opsins (OPN1MW gene) peak near 543 nm. These genes are in tandem on chromosome Xq28, and frequent recombination events result in hybrid genes that shift the L pigment’s sensitivity closer to M, often between 540 and 550 nm. Protanopia (complete L-cone dysfunction) causes a more pronounced shift, while protanomaly (partial L-cone dysfunction) results in a milder shift. Both conditions impair RG color discrimination [24,25]. Protanopia is inherited in an X-linked recessive manner, affecting approximately 1% of males and 0.01% of females [26].

Although RG CVD is the most common single-locus genetic disorder in humans and exists on a wide spectrum of severity [27], it is often treated as an all-or-nothing diagnosis. Ishihara plates [11], CAD, the Cambridge Color Test, the FM-100, the HRR Test [28], and the anomaloscope are highly accurate in detecting RG CVD. While CAD, the Cambridge Color Test, and the anomaloscope may quantify CVD, they are lengthy, difficult to interpret, and costly, especially compared to the CCT (Table 1).

The Rabin CCT accurately detects and quantifies protan deficiency. In a study of 1446 pilot applicants, it displayed 100% sensitivity and specificity in distinguishing protan-deficient individuals from trichromatic individuals, as validated by anomaloscope results. A score of ≥75 in each cone was required for normal color vision, as this threshold—two standard deviations below the normal mean on the CCT—captured 95% of individuals with typical color vision. The CCT accurately identified protan CVDs, showing greater reductions in L-cone scores, with consistent results over multiple trials. In contrast, a PIP battery (Dvorine, SPP2, F2) had sensitivities ranging from 40% to 68% [7].

**Figure 2:**
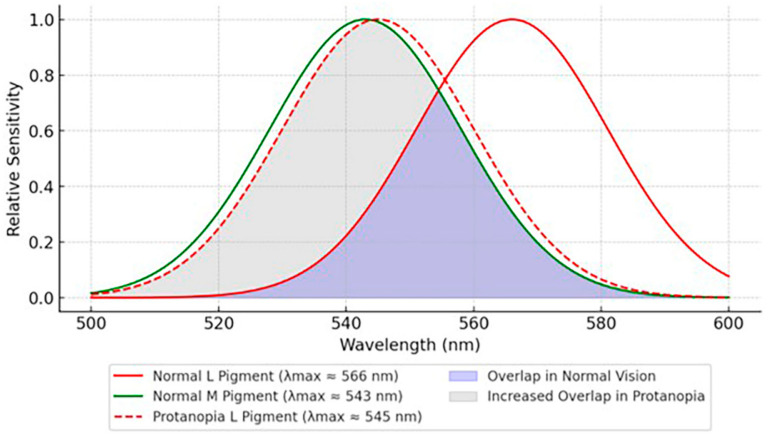
Shifted spectral sensitivity curve of the L-cone pigment in individuals with protanopia. The curve for protanopia shows a significant shift toward shorter wavelengths, reflecting the altered perception of red wavelengths and the absence of normal RG color discrimination.

It can be argued that the single-cone modulation model in the CCT may be less effective in CVD due to shifts in the standard observer function. In dichromats, L-cone and M-cone responses become more similar, making it difficult to isolate L-driven cone function accurately (Figure 2). Thus, L-cone function may be affected by M cones and vice versa. However, this does not diminish the effectiveness of the CCT. Anomaloscope spectral shifts correlate with cone-driven contrast sensitivity scores, and participants with lower CCT scores exhibit higher error rates on hue discrimination tasks and PIP testing. Nevertheless, those utilizing the CCT should be mindful of factors influencing cone scores [7].

Visually demanding occupations, such as aviation and train driving, often require normal trichromatic color vision. However, traditional color vision tests frequently misclassify individuals [29], underscoring the need for more reliable and standardized screening methods. Barbur and Rodriguez-Carmona in 2017 proposed a more nuanced classification system for color vision, introducing the categories “normal”, “functionally normal”, “safe”, “poor”, “severe”, and “supernormal” based on CAD Test performance [21]. While the CAD Test improved classification accuracy, highly correlating with the CIE 143:2001 standard (τ = 0.81, *p* < 0.001), its cost and time requirements (Table 1) restrict its use for rapid occupational screening. This makes the CCT a more accessible and efficient alternative for task-specific assessments.

#### 3.1.2. Deuteranomalous Trichromats (M-Cone Defect)

Deuteranopia is an X-linked recessive disorder caused by pathogenic variants in the *OPN1MW* gene, leading to the absence of M cones [24]. In contrast, deuteranomaly results from functionally abnormal M cones, shifting spectral sensitivity toward L cones. Together, these conditions account for the majority of congenital CVDs, with deuteranomaly being the most prevalent, affecting approximately 5% of males (Table 2). Since most individuals with CVD are anomalous trichromats rather than dichromats, accurately distinguishing between these conditions is crucial for clinical assessment and occupational screening.

While deuteranopia can be reliably detected by traditional color vision tests [30], most tests cannot accurately classify deuteranomalous trichromacy. Affected individuals may pass anomaloscope thresholds, with only slight deviations from normal [31]. Similarly, the FM100, Ishihara plate, and D15 tests have limited sensitivity in detecting mild deuteranomalous deficiencies [32,33].

The CCT has proven effective in classifying deuteranomalous individuals. In one case, the CCT detected reduced green scores (65 and 70), while the anomaloscope showed a normal midpoint and matching range. The diagnosis was later confirmed by a failed Ishihara Test (7/14 correct). The anomaloscope’s unreliability was further displayed by a study in which 15% (7/47) of cases exhibited expanded matching ranges, diminishing the diagnostic value of midpoint shift. Moreover, there was no significant correlation between the CCT scores and anomaloscope midpoint shifts in those individuals (r = 0.12; *p* > 0.26). However, as the cohort included only one deuteranomalous trichromat, further testing is needed to validate the diagnostic utility of the CCT in this subgroup [7].

#### 3.1.3. Tritanopia/Tritanomaly (S-Cone Defect)

Tritanopia, a YB autosomal dominant CVD, is caused by pathogenic variants in the *OPN1SW* gene on chromosome 7, which encodes the S-cone opsin. The condition is most associated with G79R and S214P amino acid substitutions [34,35]. Tritan defects are most often acquired, as the congenital form is rare (0.008% prevalence) [23]. Affected individuals have difficulty distinguishing between blue and green, and yellow from red, which can impact tasks such as identifying wiring in electrical work, interpreting medical images, and performing graphic design.

Traditional color vision tests, including the anomaloscope, are less effective in classifying YB defects. To address this limitation, an advanced diagnostic system for tritanopia based on the MacAdam Ellipse was proposed in 1988. This system assesses subtle color discrimination by presenting a central color stimulus and determining the MacAdam Ellipse—the range of colors that the patient perceives as indistinguishable from the reference hue [36].

The MacAdam Ellipse has been used to assess tritanopia severity in Thyroid-Associated Orbitopathy (TAO) as an early marker for Dysthyroid Optic Neuropathy (DON), a sight-threatening complication. In this study, tritanopia was considered pathological if it exceeded a threshold of 8%. Forty-seven of forty-eight DON patients exceeded this threshold. Notably, tritanopia severity did not consistently correlate with visual field (VF) defects or best-corrected VA (BCVA), despite BCVA being a key diagnostic criterion. These findings suggest that tritanopia may reveal early optic neuropathy unapparent in traditional acuity tests [37].

Several studies have linked tritan deficiency to Parkinson’s disease (PD) and Leber congenital amaurosis (LCA). PD often causes significant deficits in color discrimination along the tritan axis, likely due to retinal dopamine depletion, which particularly affects S cones [38]. Similarly, in LCA, tritan axis color discrimination is severely impaired, suggesting early S-cone dysfunction as a hallmark of the disease [39].

The CCT is reliable in quantifying tritan deficits and may be used as an adjunct tool in TAO, PD, and LCA to monitor disease progression. A study using the Rabin CCT found that glaucoma patients (*n* = 27) had significantly lower M- and S-cone CCT scores (*p* < 0.05) compared to controls (*n* = 27), with scores correlating with VF mean deviation and macular ganglion cell/inner plexiform layer (GCIPL) thickness [16]. While the Cambridge Color Test has accurately classified tritan deficiency [39], this was based on a small sample size (*n* = 7), and its findings were corroborated with the HRR Test, despite the HRR Test’s known limitations in diagnosing YB deficiencies [30]. Additionally, the test provides scores in the form of discrimination ellipses, which are more complex to interpret than the CCT’s numerical system.

#### 3.1.4. Blue-Cone Monochromacy (L- and M-Cone Defect)

Blue-cone monochromacy (BCM) is an X-linked recessive disorder caused by pathogenic variants in the *OPN1LW* and *OPN1MW* genes. The upstream locus control region (LCR) may also be affected. As a result, individuals with BCM only have functional S cones. Affected individuals cannot distinguish red or green and often experience nystagmus, myopia, photophobia, and low VA [40].

BCM is diagnosed using a combination of color vision tests, genetic analysis, and electroretinography (ERG). A study examining BCM phenotypes across three families found that older generations exhibited tritan color vision loss, eventually resembling rod monochromacy. Color vision was assessed using the HRR, D-15, and anomaloscope tests, which detected residual color discrimination but could not precisely quantify the degree of impairment [40]. The Cambridge Color Test has also quantified age-related declines in tritan sensitivity among individuals with BCM after age 30 [41,42].

While BCM has not been evaluated using the CCT, its lower cost and ease of use offer greater accessibility. Additionally, comparing age-related cone function decline to that seen in BCM may distinguish disease-related changes from normal aging, guiding adaptive strategies to mitigate visual decline in BCM patients.

The primary goal for BCM gene therapy is to reactivate dormant or enhance existing L/M cones. One therapy in development, ADVM-062, is a gene augmentation product designed to express human L-opsin [43]. In animal studies, ADVM-062 treatment enhances L-opsin expression in foveal L/M cones, extrafoveal L/M cones, and some S cones. This suggests two potential mechanisms for long-wavelength signal transmission: reactivating silent L/M cones or converting S cones into broader-spectrum photoreceptors [44].

In gene therapy, the CCT can precisely measure L- or S-cone improvements. However, expanded S-cone sensitivity could confound results by making it appear as though L- and M-cone functions are improving when, in reality, S cones are responding to a broader range of wavelengths. To distinguish between these effects, incorporating additional tests, such as electrophysiology, could help determine whether the therapy is truly reactivating L cones or merely enhancing S-cone function.

#### 3.1.5. Achromatopsia (Total Color Blindness)

Achromatopsia (ACHM) is an autosomal recessive disorder characterized by a complete inability to perceive colors, affecting approximately 1 in 30,000 people. The condition is primarily caused by pathogenic variants in the *CNGA3* and *CNGB3* genes, which encode the alpha and beta subunits of the cone cyclic nucleotide-gated channel, respectively. These variants disrupt the entire phototransduction cascade, a process essential for converting light into electrical signals in the retina, rather than affecting specific photoreceptors, as seen in RG and YB CVDs. Less common causes of ACHM include pathogenic variants in *ATF6*, *GNAT2*, *PDE6C*, and *PDE6H*, which also play roles in phototransduction [45]. Individuals with ACHM may concomitantly experience severe photophobia, nystagmus, VF defects, and reduced VA.

Initial ACHM evaluation included VA, nystagmus, and color vision testing. However, conventional color vision tests are often unreliable in ACHM, as patients may distinguish colors based on brightness variations or learned associations rather than true color perception [46]. Additional diagnostic tools include optical coherence tomography (OCT), fundus autofluorescence, ERG, and genetic testing. Management focuses on symptom relief, such as tinted lenses for photophobia and assistive tools like “Color Quest” for color guidance [47]. Early clinical trials for *CNGA3* and *CNGB3* gene replacement have shown promising results [48].

Other conditions with ACHM-like symptoms include BCM, Alström syndrome, cerebral ACHM, LCA, and bradyopsia. In BCM, differential diagnosis relies on peak luminosity testing and ERG, where BCM patients exhibit cone responses near 400 nm (S cones), whereas ACHM patients show only rod responses. Alström syndrome is distinguished by additional features, such as obesity, hearing loss, and cardiomyopathy [49]. Cerebral ACHM results from brain injury or infection to V4 of the ventral occipital cortex. Unlike inherited ACHM, it often presents with neurological symptoms such as visual agnosia, prosopagnosia, or spatial disorientation [48]. LCA is characterized by severe visual impairment from infancy, poor color discrimination, and night blindness, caused by pathogenic variants in genes such as *AIPL1*, *CABP4*, *CEP290*, *GUCY2D*, and *RPGRIP1*, which are involved in photoreceptor development, maintenance, and function [50]. Finally, bradyopsia, a condition characterized by delayed cone response, mimics ACHM but results from a pathogenic variant in the *RGS9* gene, which plays a key role in the phototransduction cascade [39,51].

The CCT can help characterize visual dysfunction in ACHM and related conditions with overlapping features. In Alström syndrome, it can reveal distinct contrast sensitivity patterns. In cerebral ACHM, the CCT can detect cone-driven function deficits that align more with central processing dysfunction rather than peripheral retinal impairment, distinguishing between the contributions of the retina and brain in color vision loss. In LCA, early S-cone deficiencies indicate a unique disease progression, differentiating it from the diffuse cone impairment seen in ACHM [39]. In bradyopsia, the CCT can assess contrast sensitivity over time, revealing a pattern that distinguishes it from ACHM. By elucidating how genetic mutations such as *RGS9* (bradyopsia) and *AIPL1* (LCA) affect vision, the CCT aids in precise diagnoses and the discovery of novel genetic causes of color vision disorders, potentially guiding the development of new therapies.

Beyond its role in differential diagnosis, the CCT may help identify residual color vision in ACHM, which could aid in pinpointing less severe genetic variants and guiding gene therapies.

### 3.2. Cone Dystrophy/Cone–Rod Dystrophy

Cone–rod dystrophy (CRD) is an inherited retinal disorder affecting approximately 1 in 40,000 individuals worldwide. It is characterized by the progressive apoptosis of cones, followed by rods, causing decreased VA, VF loss, and CVD [52]. The four genes most implicated in CRD pathogenesis are *ABCA4*, *CRX*, *GUCY2D*, and *RPGR*. Autosomal recessive mutations in *ABCA4* account for 30–60% of cases, disrupting the exocytosis of toxic retinoid byproducts from photoreceptors [53]. Autosomal dominant mutations in *CRX* and *GUCY2D* impede photoreceptor differentiation and phototransduction regulation, respectively [54,55]. Finally, X-linked recessive mutations in *RPGR* disrupt photoreceptor ciliary function [56].

ERG is essential for diagnosing CRD, detecting early fundoscopic lesions and reduced cone ERG responses even in asymptomatic patients. Repeat ERGs after 1 to 2 years are conducted to monitor symptom progression. While genetic testing for *ABCA4*, *CRX*, *GUCY2D*, and *RPGR* can confirm the diagnosis, accessibility remains limited due to cost, availability, and the complexity of genetic variations. There is no cure for CRD. Management focuses on slowing photoreceptor degeneration and addressing complications. Vitamin supplements and light protection help preserve ocular health, while surgery can treat cataracts and macular edema. By the age of 30, most patients experience legal blindness, necessitating professional counseling. Gene therapy research is challenged by the genetic heterogeneity of CRD, underscoring the need for further studies to identify key pathogenic pathways [57].

Although cone dysfunction is well documented in CRD and color vision testing can detect associated deficits, it is rarely utilized in diagnosis and management [58]. Yet, such testing can help differentiate genetic variants. For example, a study of five CRD patients with *KCNV2* variants showed preserved L-cone responses until late stages [59]. By contrast, *CNGA3*-related CRD often presents with early RG defects, and *RHO* mutations cause gradual cone decline [60]. Integrating color vision testing into clinical practice could aid in identifying genetic variants without genetic testing.

The CCT may serve as an early functional marker of cone dysfunction in suspected CRD cases. Clinically, significant or rapidly progressing CVD might prompt earlier referrals for low-vision aids or vocational counseling or prioritize patients for gene therapy trials. In research, the CCT could serve as an outcome measure, allowing investigators to correlate changes in color vision with visual function and disease biomarkers.

### 3.3. Diabetic Retinopathy

Diabetic retinopathy (DR), a complication of diabetes, causes retinal vascular damage, ischemia, neovascularization, and macular edema. In the United States, DR cases are projected to reach 16 million by 2050, with 3.4 million at risk of blindness. Early detection is crucial to prevent irreversible damage, using fundus exams, fundus photography, and AI-assisted imaging like AEYE-DS, EyeArt AI, and LumineticsCore [61].

While current diagnostic techniques effectively identify vascular changes, they may miss early neuroretinal dysfunction [62]. Psychophysical tests, such as contrast sensitivity exams, dark adaptometry, and color vision assessments, may help identify these early functional impairments. Although VA is commonly used in clinical evaluations, it does not reliably reflect retinal health, highlighting the need for complementary functional assessments [63,64].

Tritan defects affect up to 49% of DR patients, with S-cone loss often preceding VA decline [65]. Studies link CVD severity to DR progression [66,67,68,69]. The Rabin CCT has detected moderate cone dysfunction in DR patients with 20/20 VA and no macular edema, demonstrating structural–functional correlations confirmed by OCT [70].

The most used color vision tests for evaluating DR, such as D15 and FM-100, are prone to test–retest variability and learning effects. Some patients show improved performance despite disease progression, likely due to familiarity with the test [71]. The CCT may address these issues by using the Landolt-C optotype, minimizing influence from prior exposure. Furthermore, it maintains consistent contrast sensitivity throughout the test, avoiding variability caused by luminance or contrast fluctuations, a common issue in older color vision tests. 

Psychophysical tools, including color vision assessments, offer valuable functional insight in DR evaluation. The CCT may be integrated into routine screening for patients with diabetes, especially those reporting vision changes without observable vascular damage. S-cone decline may prompt earlier intervention, such as optimizing glycemic control or initiating DR treatment. However, further clinical trials are necessary to validate the CCT’s role in detecting early dysfunction and complementing existing diagnostic tests.

### 3.4. Macular Disorders

#### 3.4.1. Age-Related Macular Degeneration

Age-related macular degeneration (AMD) is a progressive retinal disease that primarily affects individuals over 50. It is classified into dry (macular atrophy) or wet (abnormal vessel growth) AMD. Diagnosis involves clinical exams and imaging. OCT can detect retinal atrophy in dry AMD and neovascularization in wet AMD, while fluorescein angiography can confirm choroidal neovascularization. Routine monitoring includes VA testing and OCT. While there is no cure for dry AMD, its progression may be slowed with AREDS/AREDS2 supplements and lifestyle changes. Wet AMD is managed with anti-VEGF injections, which prevent significant vision loss in most patients [72].

AMD disrupts cone photoreceptors through opsin redistribution, axonal anomalies, and degeneration [73]. These structural changes impair the transmission of color and contrast information, often leading to functional deficits before significant VA loss becomes evident [74].

The Cambridge Color Test, CAD, and the CCT have been used to evaluate color vision in AMD patients. The CCT identified M- and L-cone-driven functional deficits in dry AMD, while some neovascular AMD (NVAMD) patients exhibited preserved S-cone sensitivity [9]. The Cambridge Color Test revealed reduced color discrimination in AMD patients but did not find differences between dry AMD and NVAMD [74]. CAD primarily detected axis deficiencies in AMD patients with reticular pseudodrusen but did not provide cone-specific details [75]. Other color vision assessments, including the FM-100 [76] and D15 [77], show limited sensitivity to CVD in AMD.

Color vision screening may be conducted in patients with early or suspected AMD, especially those without significant VA loss. A decline in M- or L-cone function may indicate disease progression. Further clinical trials with larger sample sizes are necessary to validate the role of the CCT in AMD management and its potential to complement existing diagnostic tools.

#### 3.4.2. Occult Macular Dystrophy

Occult Macular Dystrophy (OMD) is a rare autosomal dominant disorder caused by pathogenic variants in the *RP1L1* gene, which is crucial for photoreceptor function. Diagnosis is challenging due to central VA loss and photophobia in the absence of visible fundoscopic abnormalities. OCT and electrophysiological testing can reveal subtle retinal abnormalities, while genetic analysis, such as identifying the *p.R45W* pathogenic variant in *RP1L1*, can confirm the diagnosis when results are inconclusive [78].

Decreased cone density is a hallmark of OMD [79], but color vision testing is not typically used in its assessment. Recent research using temporal contrast sensitivity (tCS) has shown that affected individuals experience impairments in L- and M-cone-driven functions. Milder disease, with preserved foveal structure on OCT, is associated with better cone function, while more severe dysfunction patterns correlate with greater visual impairments, myopia, and OCT structural changes [80].

While tCS is a specialized and costly research tool, this study underscores the importance of psychophysical tests in diagnosing and monitoring OMD. More accessible tests like the CCT could be valuable for clinical use. By integrating the CCT into OMD testing, physicians may effectively track disease progression. The CCT may also be used as a functional measure in OMD research, which currently focuses on VA [81].

### 3.5. Optic Neuritis

Optic neuritis (ON) is an inflammatory condition involving the autoimmune degradation of the optic nerve’s myelin sheath, often associated with autoimmune disorders or viral infections. It typically presents in young adults with sudden monocular eye pain and significant vision loss, followed by dyschromatopsia and contrast sensitivity deficits. ON is strongly associated with multiple sclerosis (MS), with about 50% of MS patients developing ON and 15–20% experiencing it as the first sign of demyelination [82]. Diagnostic testing for ON includes BCVA, VF analysis, and OCT to assess RNFL thinning. Magnetic resonance imaging (MRI) may help identify optic nerve demyelinating lesions, aiding in the diagnosis of MS [82]. The ON Treatment Trial (ONTT) established that ON is managed with intravenous methylprednisolone (500–1000 mg for 3 days), followed by an oral prednisone taper (1 mg/kg daily) [83]. While visual symptoms typically improve within three months, some patients continue to experience contrast sensitivity dysfunction (measured by the FM-100), even with 20/20 VA [84,85].

Impairment in color perception is well documented during and after ON. Parvocellular fibers, which are responsible for color perception and fine visual detail, are particularly vulnerable to demyelination and axonal loss in ON due to their smaller axonal diameter [86]. Additionally, histopathologic studies of MS have demonstrated selective injury to ganglion cells in the macular region, further supporting the vulnerability of these fibers in ON [87].

A study of 75 patients with monocular ON assessed cone contrast sensitivity, RNFL thickness, and macular volume (MV). Using the Rabin CCT, the researchers observed significantly lower cone contrast scores in the affected eye, correlating with RNFL thickness and MV. Additionally, CVD was also detected in the fellow eye, suggesting subclinical bilateral dysfunction [88]. Furthermore, the CCT may detect residual disease activity in MS and post-ON recovery. A recent study found that MS patients, regardless of optic neuritis history, had significantly reduced M- and S-cone scores compared to healthy controls, even with normal RNFL thickness and VA [9].

These findings suggest that the CCT may detect persistent functional deficits in ON more sensitively than VA or OCT alone. We suggest incorporating the CCT at the time of ON diagnosis, alongside VA, OCT, and MRI, to establish baseline deficits in cone-specific contrast sensitivity and correlate findings with RNFL thickness and optic nerve lesions. Additionally, because the CCT has detected functional deficits in the fellow eye of ON patients, testing can extend to the unaffected eye to monitor for subclinical disease activity, particularly in those with MS. Regular CCT assessments (biannually or annually) could serve as an early biomarker of neurodegeneration, guiding treatment before VA loss. Persistent CCT deficits despite normal VA and OCT may indicate a higher risk of future demyelination, warranting closer neurological follow-up.

### 3.6. Glaucoma

Glaucoma is a group of optic neuropathies characterized by elevated intraocular pressure (IOP), leading to retinal ganglion cell loss, optic nerve head cupping, and irreversible vision loss [89]. Primary open-angle glaucoma (POAG), the most common subtype, remains asymptomatic until significant damage occurs, underscoring the need for early detection [90]. Diagnosis involves OCT and VF testing. Together, these tests assess optic nerve damage and guide management decisions [91].

CVD in glaucoma has been recognized since 1883 [92]. In early-stage POAG, YB discrimination is primarily affected, while advanced disease can lead to RG deficiency due to selective retinal ganglion cell damage [93]. It is hypothesized that the unique morphology of S-cone neuronal connections causes them to be more vulnerable to elevated IOP [94]. Notably, CVD may emerge before detectable changes in HVF testing or OCT imaging, suggesting its potential as an early biomarker for glaucoma-related impairment [95,96].

A study utilizing the CCT in glaucoma patients found significantly lower M-cone and S-cone contrast thresholds compared to controls, with strong correlations to HVF mean deviation, GCIPL thickness, and findings from previous studies [16,93]. CAD identified significantly greater RG than YB deficiency in mild glaucoma [97], contrasting with several studies that primarily implicate YB deficiency in mild glaucoma [92,98]. This discrepancy suggests that while CAD is effective in detecting chromatic dysfunction, it may have limitations in fully capturing the extent of YB impairment in glaucoma. Consequently, the CCT may provide a more comprehensive evaluation of cone-specific dysfunction.

At diagnosis, the CCT may be performed alongside standard assessments such as OCT and HVF testing to assess early functional impairment. Since CVD may precede structural damage, the CCT could serve as an adjunctive biomarker for detecting early glaucomatous changes. During routine follow-ups, repeated CCTs can track disease progression, especially in patients with stable HVF and OCT findings but persistent functional complaints. If significant CCT deficits emerge despite stable conventional metrics, clinicians may consider more aggressive IOP-lowering strategies or closer monitoring.

### 3.7. Brain Injury

#### 3.7.1. Traumatic Brain Injury

Traumatic brain injury (TBI) is a leading cause of both mortality and long-term disability in the United States [99]. Primary causes include falls, sports injuries, and motor vehicle accidents. These events result in axonal shearing, ischemic damage, and secondary neuroinflammation. TBI is classified as mild, moderate, or severe based on structural imaging, the Glasgow Coma Scale, and outcomes such as the duration of loss of consciousness [100]. Mild TBI (concussion) is often more insidious than moderate or severe cases, as its subtle abnormalities may not be detectable on neuroimaging, yet it can still result in persistent neurological dysfunction [101]. While immediate brain damage caused by TBI is often permanent, secondary injury that develops over time may be preventable with therapeutic intervention [102].

Initial TBI assessment is conducted in acute care settings, involving a thorough clinical evaluation of neurological status and cervical spine assessment. Follow-up care includes neuroimaging, serological tests, and the monitoring of intracranial pressure to prevent secondary brain injury [103]. Rehabilitation depends on the cognitive sequelae of the injury and is managed by a multidisciplinary team, which may include physical therapists, speech pathologists, and neurologists [104].

Early neurorehabilitation has been shown to improve outcomes in TBI, leading to less long-term brain damage, better functional recovery, and shorter hospital stays, especially in moderate-to-severe cases [105,106,107]. Despite these benefits, one-third of mild TBI patients with vision issues delay evaluation for up to two years [108]. While visual dysfunction is one of the most common complaints among TBI patients [109], it often goes undiagnosed until formal vision screening [110,111]. However, many rehabilitation settings still lack ophthalmologists or optometrists, despite the risks associated with delayed intervention [112].

Integrating visual assessments, such as the CCT, into TBI care can help localize brain damage and guide rehabilitation strategies. Color vision tests may detect neurological impairment before overt symptoms emerge [113]. Since color perception can reflect neural disruptions rather than ocular pathology, these tests are particularly useful in the acute phase of the injury. Even mild TBI patients without visual complaints show measurable deficits, including reduced contrast sensitivity and structural brain changes [114]. Similarly, OCT has revealed RNFL thinning in TBI patients without overt symptoms, underscoring its potential as an early biomarker of neural damage [115].

The CCT can help identify affected visual processing pathways, aiding in the diagnosis of specific brain lesions related to TBI. For instance, lesions in the ventral visual stream, located in the occipital and parietal lobes, can cause chromatic discrimination deficits. Even when structural imaging such as OCT shows no significant damage, CVD can still indicate neural disruptions [116]. The management strategies for CVD include the use of colored glasses to alleviate photophobia [117] and vision therapy to improve binocular vision, ocular motility, and visual attention, enhancing spatial awareness and reading speed [118]. Early detection and treatment using neuroprotective agents like P7C3-S243 can prevent chronic visual deficits by protecting retinal ganglion cells [119]. Integrating the CCT into TBI evaluation can help mitigate damage and improve functional recovery.

Color vision testing shows significant merit in adjunctive evaluation and monitoring [120], and the CCT particularly offers a rapid, reliable assessment.

#### 3.7.2. Non-Traumatic Brain Injury

Color vision testing can detect early visual deficits in neurologic conditions such as strokes, brain tumors, and cognitive impairments. In patients with posterior cerebral artery (PCA) stroke, 22% exhibited CVD despite normal VF tests [113]. Mid-range visual deficits, including color vision impairment, often occur following stroke, sometimes without other visual disturbances [121]. These deficits affect daily activities like reading and navigation, underscoring the importance of early detection. Incorporating color vision assessments into post-stroke rehabilitation could help clinicians gain a deeper understanding of visual dysfunction and customize interventions for improved outcomes.

Color vision testing can also assess visual dysfunction in patients with brain tumors. Lesions in the ventral occipital–temporal pathways, such as those caused by gliomas or metastatic tumors [122], can lead to cerebral achromatopsia due to damage to color processing centers [123]. As tumors progress, subtle visual deficits may worsen, and color vision testing offers a non-invasive method to monitor disease progression and treatment response. Post-treatment assessments following tumor resection or radiation therapy can provide valuable insights into functional recovery.

Recent research underscores the role of retinal changes in cognitive impairment (CI), expanding the scope of visual function testing in brain injury assessments [124]. A pilot study of 69 patients found altered retinal microvascular networks and functional deficits, such as color vision impairment, in those with CI [125]. These findings suggest retinal vascular and functional parameters as potential biomarkers for cognitive decline. Integrating both structural and functional assessments may enhance sensitivity in detecting brain lesions and tracking recovery, with color perception testing offering valuable insights into the integrity of visual processing pathways.

### 3.8. Drug Toxicity

Acquired CVD can result from drug toxicity, particularly from chloroquine (CQ)/hydroxychloroquine (HCQ), digoxin, ethambutol (EMB), and PDE-5 inhibitors [126]. CQ/HCQ can cause optic neuropathy and retinopathy, leading to early tritan defects that progress to RG defects. Digoxin induces temporary RG defects by inhibiting Na+/K+ ATPase in retinal cells. EMB is associated with YB dyschromatopsia due to optic neuropathy, while PDE-5 inhibitors like sildenafil cause transient blue-tinted vision by affecting cone cell phototransduction.

CQ and its less toxic derivative, HCQ, are used to treat malaria, autoimmune diseases (lupus, rheumatoid arthritis), and, in some cases, COVID-19 [127,128]. CQ/HCQ-induced CVD likely results from drug binding to melanin in the retinal pigment epithelium (RPE), causing toxic accumulation, disrupted metabolism, and photoreceptor loss, leading to bull’s eye maculopathy and CVD [129]. Studies using color vision tests like Ishihara, D-15, HRR, and City University tests have shown varying results. One study found CVD in only a part of patients with bull’s eye maculopathy [130], while another reported that twenty-eight of thirty CQ retinopathy patients failed at least one test [131]. Traditional tests often lack sensitivity for early detection, a limitation that quantitative tests like the CCT can address [7,17].

Digoxin, a cardiac glycoside used to treat heart failure and arrhythmias, is linked to CVD through its effect on the Na+/K+ ATPase pump in photoreceptors, Müller cells, and the RPE [132]. Up to 80% of patients report transient, reversible CVD [133], often signaling digoxin toxicity at high levels [134]. While studies show mixed results, one study found a higher prevalence of severe tritan defects in digoxin users [135].

EMB, a drug for the treatment of tuberculosis, is linked to acquired CVD, primarily affecting YB perception [136]. Its mechanism involves zinc chelation, excitotoxicity, and mitochondrial dysfunction of the optic nerve [137,138]. Kaimbo et al. found that CVD emerged after five months of EMB use, with some cases progressing to complete color blindness, although most patients recovered following treatment cessation [139].

PDE-5 inhibitors like sildenafil, vardenafil, and tadalafil, used to treat erectile dysfunction, can cause temporary YB CVD by inhibiting PDE-6, an enzyme crucial for retinal phototransduction. While some studies report no significant effects on color vision [140], others find notable CVD [141]. Most defects are reversible, with higher doses increasing the risk. However, study limitations, including small sample sizes, qualitative color vision testing, and short follow-up periods, underscore the need for long-term research with standardized assessments.

Quantitative color vision testing can assist future drug development by identifying properties that reduce the risk of acquired CVD. Additionally, incorporating the CCT or similar color vision assessments in patient monitoring may aid in the early detection of drug toxicity. Given the widespread use of these medications, improving CVD management could have a significant impact on addressing broader concerns related to ocular toxicity.

## 4. Discussion and Conclusions

The CCT stands out as a cost-effective tool for color vision assessment, offering rapid administration (3 to 6 min) while maintaining high diagnostic accuracy. It is accessible in clinical settings equipped with computer-based testing, allowing for efficient use in high-volume clinics. The CCT offers superior sensitivity and specificity, making it useful for diagnosing and monitoring both inherited and acquired CVD. It is valuable in occupational screening, where precise severity classification is necessary to determine job eligibility. Moreover, the CCT has applications in neuro-ophthalmology. In conditions like DR, ON, glaucoma, TBI, and stroke, early CVD detection can provide insight into underlying neural disruptions. Acquired CVD from drug toxicity can be effectively screened and monitored with the CCT as well. Table 3 summarizes the proposed use and performance metrics of the CCT in all conditions discussed in this article.

The CCT has its limitations. One study reported a decline in specificity in individuals over 65, although this finding was not replicated in another study [17,142]. Additionally, single-cone modulation models may not fully isolate photoreceptor function. Due to the dense packing of cones, stimuli often engage multiple cones, and even with targeted stimulation, perceptual variability arises because the brain processes the combined signals from all cone types. Furthermore, the CCT also relies on differential contrast sensitivity across cone types, but in dichromats and anomalous trichromats, neural compensation may alter spectral sensitivity, complicating the precise isolation of cone function.

Like other computer-based color vision tests, the CCT may not fully capture color discrimination in natural environments. Most data generated by these tests are pixel-based rather than object-based, which limits their ability to reflect color vision functions in real-world viewing conditions [143]. However, this limitation applies to all computer-based color vision tests, not just the CCT.

Ultimately, while the CCT remains a practical and efficient tool for color vision assessment, its limitations underscore the broader challenge of defining and quantifying color perception. Integrating knowledge of opponent processing, cone interactions, and luminance perception into test development could enhance its accuracy. Incorporating the CCT into routine practice supports early detection, personalized treatment strategies, and more equitable occupational assessments, reducing unnecessary job restrictions while maintaining safety standards.

## Figures and Tables

**Table 1:** A summary of color vision tests by goal, time, cost, sensitivity, and specificity. All metrics are approximations.

Name	Time to Complete (Minutes)	Cost	Sensitivity	Specificity
Detect				
Ishihara plates [11,12]	2–4	USD 550 *	98.4%	100%
HRR [11,12,13]	5–10	USD 610 *	87–98%	33–100%
Classify				
Anomaloscope	15–20	USD 18,000	100%	100%
FM-100 [12,14]	10–15	USD 1150 *	81.3–100%	93–95.4%
D15 [15]	2–10	USD 550 *	58%	100%
Quantify				
CAD [14]	12–30	USD 9000	100%	100%
Cambridge Color Test	8–15	USD 30,000 **	***	***
CCT [5,7,16,17]	4–6	USD 4000 to 10,000	100%	100%

* Includes CIE Illuminant D65 price (~USD 350). ** Includes all three variations of the Cambridge Color Test. *** Data not available.

**Table 2:** A summary of the different forms of congenital color vision deficiency [23].

Deficiency	Cone(s) Affected	Inheritance	Prevalence
Anomalous trichromacy			
Protanomaly	Red	XLR	1.08%
Deuteranomaly	Green	XLR	4.63%
Tritanomaly	Blue	AD	See tritanopia *
Dichromacy			
Protanopia	Red	XLR	1.01%
Deuteranopia	Green	XLR	1.27%
Tritanopia	Blue	AD	1 in 500
Monochromacy			
Green-cone monochromacy	Red and blue	Dual XLR and AD ^#^	≤1 in 1,000,000
Red-cone monochromacy	Green and blue	Dual XLR and AD ^#^	≤1 in 1,000,000
Blue-cone monochromacy	Red and green	XLR	1 in 100,000
Rod monochromacy and incomplete achromatopsia	Red, green, and blue	AR	1 in 33,000–50,000

AD: autosomal dominant; AR: autosomal recessive; XLR: X-linked recessive. Prevalence estimates are based on European populations. For X-linked recessive conditions, prevalence refers specifically to males. * Tritanomaly is considered a phenotypic variation of tritanopia, and their combined prevalence may be as high as 1 in 500. ^#^ Red- and green-cone monochromacy could theoretically result from the combined inheritance of deuteranopia and tritanopia or protanopia and tritanopia, respectively. However, all documented cases have exhibited some degree of post-receptoral deficit.

**Table 3:** Summary of CCT’s use and performance metrics for all conditions discussed.

Condition	Proposed CCT Use	Performance Metrics
Congenital CVDs	Diagnostic tool	Large-scale validation studies with anomaloscope; 100% sensitivity/specificity
CRD	Monitoring progressive cone dysfunction	*
Glaucoma	Detection of early S-/M-cone loss	Scores correlate with OCT and VF loss
DR	Detection of early neuroretinal dysfunction	Scores correlate with OCT [70]
AMD	Functional assessment alongside OCT	Scores correlate with OCT and gradings by retinal specialists [9]
OMD	Functional confirmation in normal-appearing retina	*
ON	Quantification of post-inflammatory changes	Scores correlate with OCT and gradings by retinal specialists [88]
MS	Detection of subclinical visual pathway involvement	Scores correlate with diagnosis by specialists [9]
Brain injury	Assessment of visual processing disruption	*
Drug toxicity	Early detection of cone dysfunction	*

* No data available.

## Data Availability

No new data were created or analyzed in this study.
